# Characterization and Migration Activity of Thermoresponsive Silk Fibroin–Aloe Vera Gel in Normal and Diabetic Fibroblasts

**DOI:** 10.3390/gels12030188

**Published:** 2026-02-24

**Authors:** Phassorn Khumfu, Witwisitpong Maneechan, Thanasorn Panmanee, Nuttapong Khiaonoi, Sukunya Ross, Gareth Ross, Céline Viennet, Jarupa Viyoch

**Affiliations:** 1 Department of Pharmaceutical Technology, Faculty of Pharmaceutical Sciences, Naresuan University, Phitsanulok 65000, Thailand; phassornk66@nu.ac.th (P.K.); witwisitpong.ma@nu.ac.th (W.M.); thanasornp66@nu.ac.th (T.P.); 2 UMR 1098 RIGHT INSERM EFS FC, DImaCell Imaging Resource Center, Marie & Louis Pasteur University, 25000 Besançon, France; 3 Department of Mechanical Engineering, Faculty of Engineering, Naresuan University, Phitsanulok 65000, Thailand; nuttapongk65@nu.ac.th; 4 Department of Chemistry, Faculty of Science, Naresuan University, Phitsanulok 65000, Thailand; sukunyaj@nu.ac.th (S.R.); gareth@nu.ac.th (G.R.); 5 Center of Excellence for Innovation in Chemistry (PERCH-CIC), Faculty of Pharmaceutical Sciences, Naresuan University, Phitsanulok 65000, Thailand

**Keywords:** silk fibroin, *Aloe vera* gel extract, thermosensitive gel, diabetic wound healing, human diabetic fibroblasts

## Abstract

Diabetic wounds remain a major clinical challenge due to delayed healing caused by chronic inflammation and impaired fibroblast activity. Here, we present a thermoresponsive gel composed of chitosan (CS) and poloxamers (POL) incorporating silk fibroin (SFB) and *Aloe vera* gel extract (AV), developed for topical application and, for the first time, evaluated using an inflammation-induced diabetic fibroblast model. The optimized formulation exhibited rapid sol–gel transition at physiological temperature and suitable rheological properties for effective wound coverage. In vitro evaluation using human normal fibroblasts (HNF) and human diabetic fibroblasts (HDF), under both basal and inflammation-induced conditions, demonstrated good cytocompatibility and a significant enhancement of fibroblast migration, particularly in an inflammatory microenvironment simulated by high glucose, lipopolysaccharide (LPS), interleukin-6 (IL-6), and tumor necrosis factor-α (TNF-α). These findings highlight the potential of the developed thermoresponsive gel as a promising biomaterial platform for improving diabetic wound healing under inflammation-relevant conditions.

## 1. Introduction

Diabetic wounds remain a major clinical complication of diabetes mellitus, characterized by delayed and often incomplete healing. These chronic wounds, particularly diabetic foot ulcers (DFUs), are associated with persistent inflammation, high susceptibility to infection, and an increased risk of amputation and mortality. The impaired healing process is driven by complex pathological mechanisms, making effective treatment both challenging and costly [1,2,3].

At the cellular level, fibroblast dysfunction plays a central role in the delayed healing observed in diabetic wounds. Fibroblasts are essential for wound repair through their involvement in extracellular matrix (ECM) deposition, collagen synthesis, and wound contraction. Under diabetic conditions, chronic hyperglycemia induces oxidative stress [4,5,6] and metabolic imbalance [5,6], leading to impaired fibroblast migration, proliferation, and ECM production [4,7]. These alterations significantly slow tissue regeneration and wound closure [8].

In addition to fibroblast impairment, dysregulated interactions between fibroblasts and immune cells further exacerbate diabetic wound pathology. Prolonged inflammatory signaling contributes to excessive production of pro-inflammatory cytokines, such as interleukins (ILs) and tumor necrosis factor-α (TNF-α), by immune cells, leading to impaired fibroblast function, including migration, which is a critical biological activity for wound healing [9,10,11,12,13,14,15,16,17,18].

Current therapeutic strategies for diabetic wound management include pharmacological treatments, conventional wound dressings, creams, and topical gels incorporating antibiotics or anti-inflammatory agents. While many commercial products address individual aspects of wound healing, such as infection control or inflammation reduction, they often lack multifunctionality. Given the multifactorial nature of diabetic wound pathology, there is a growing need for advanced wound care products that combine physical protection with bioactive and biochemical support to promote coordinated healing.

In this context, bioactive and multifunctional wound care gels based on natural biopolymers have gained increasing attention. Silk fibroin (SF), derived from *Bombyx mori*, exhibits excellent biocompatibility [19], mechanical strength [20], and structural versatility [21]. Numerous studies have demonstrated that SF supports fibroblast adhesion, migration, and differentiation [22], thereby enhancing ECM formation and tissue regeneration. These properties make silk fibroin an attractive scaffold material for wound-healing applications.

*Aloe vera* (*Aloe barbadensis* Miller) is another widely studied natural bioactive agent with well-documented wound-healing properties [23,24,25,26]. Its polysaccharides, glycoproteins, and flavonoids exhibit anti-inflammatory, antibacterial, and antioxidant activities. *Aloe vera* has been shown to modulate cytokine expression, stimulate fibroblast activity, and accelerate re-epithelialization [27]. Previous in vitro, in vivo, and clinical studies have reported enhanced wound closure when fibroin-based formulations were combined with *Aloe vera* extracts, particularly in diabetic wound models [28,29,30].

Beyond bioactivity, an effective wound care product must also possess appropriate physicochemical and handling properties. Thermosensitive gel systems based on poloxamers have emerged as promising platforms due to their sol-to-gel transition at physiological temperature, enabling easy application and prolonged retention at the wound site [31,32]. Incorporating bioactive polymers and plant extracts into such systems may further enhance their therapeutic potential.

This study extends our previous work [33] by developing and optimizing a thermoresponsive silk fibroin–*Aloe vera* extract gel and evaluating its performance in an inflammation-induced diabetic fibroblast model, providing insight into its potential for diabetic wound applications [34,35,36].

Therefore, the present study aims to develop and characterize a thermosensitive gel incorporating silk fibroin/*Aloe vera* for diabetic wound healing. The designed formulations were evaluated in terms of their physicochemical properties and biological performance. In vitro assessments were conducted using human normal fibroblasts (HNF) and human diabetic fibroblasts (HDF) under normal and diabetic-mimicking inflammatory conditions by high glucose and pro-inflammatory cytokines to elucidate the effects of the gel formulation on wound-healing-related responses [34,35,36,37,38,39,40,41].

## 2. Results and Discussion

To highlight the novelty of this work, a thermoresponsive gel incorporating silk fibroin (SFB) and *Aloe vera* gel extracts (AV) was formulated, building upon previously reported chitosan-based hydrogels and SFB/AV films [31,32,33,37,42,43,44]. In contrast to earlier formulations that primarily investigated a single polymer ratio or a narrow temperature range, the present gel combines a specific SFB/AV/CS ratio with poloxamer 188 and 407, and β-glycerophosphate, enabling rapid and stable thermoresponsive gelation suitable for topical wound application. Additionally, this study integrates rheological optimization with biological evaluation under diabetic-mimetic fibroblast conditions, which has rarely been addressed in prior reports. Previous hydrogels have reported either rheological properties [31,32] or mechanical assessments [37], but the combined consideration of viscoelasticity, network integrity, and cell viability under pathophysiological conditions distinguishes this system. Moreover, this work focuses on clinically relevant temperature behavior (including 37–40 °C), ensuring that gel performance is maintained under both physiological and hyperthermic conditions, which is critical for effective wound coverage. These aspects collectively demonstrate the novelty and translational potential of the developed thermoresponsive gel formulation.

### 2.1. Characteristics of Silk Fibroin and Aloe vera Gel Extracts

**Figure 1:**
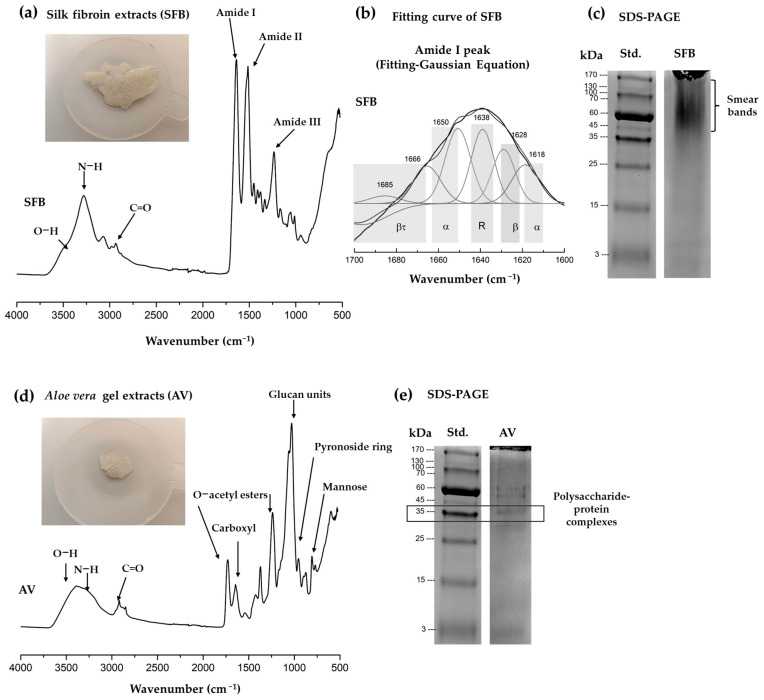
(**a**) FTIR spectrum of silk fibroin (SFB) extract; (**b**) Fitting curve of the amide I band showing β-sheet and amorphous phases (β_t_; β-turn structure, R; random coil structure, α; α-helix β; β-sheet structure) determined by Gaussian fitting; (**c**) Molecular weight distribution of proteins in SFB analyzed by SDS-PAGE, exhibiting a smear band and partial retention; (**d**) FTIR spectrum of *Aloe vera* gel extracts (AV); and (**e**) Molecular weight distribution of proteins in AV extract by SDS-PAGE.

Silk fibroin (SFB) and *Aloe vera* gel extracts (AV) were characterized to ensure reproducibility and quality control of the bioactive components. Silk fibroin protein content was determined as ≥90%, whereas *Aloe vera* gel extracts contained ≥4% protein, consistent with our previous reports [33]. The protein contents of silk fibroin (SFB) and Aloe vera (AV) were 93.00 ± 2.00% and 7.00 ± 0.67%, respectively (Table 1). The FTIR spectrum of silk fibroin extracts (Figure 1a) exhibited distinct absorption bands corresponding to amide I (~1650 cm^−1^), amide II (~1530 cm^−1^), and amide III (~1230 cm^−1^), which are characteristic of protein secondary structures [30,33]. The broad amide I band indicated the coexistence of β-sheet conformations (~1620–1640 cm^−1^) and random coil structures (~1640–1650 cm^−1^) [24,25,26,27,28,29,30]. The β-sheet structures are known to provide mechanical stability and strength to fibroin-based biomaterials [30,33], whereas random coil regions contribute to chain flexibility. This combination of ordered and disordered structures highlights the structural organization of silk fibroin, which is essential for its biomaterial properties [24,33].

SDS-PAGE analysis of silk fibroin (Figure 1c) indicated partial retention of protein structure, although the presence of smear bands suggested some degree of degradation in the range of 35–170 kDa. This phenomenon may be attributed to the extraction and preparation steps, such as the use of calcium chloride or shear forces during dialysis.

The FTIR spectrum of *Aloe vera* gel extract (Figure 1d) displayed characteristic polysaccharide and bioactive component signals. Key features include a broad O–H stretching band around 3400 cm^−1^, C–H stretching near 2920 cm^−1^, a prominent C=O stretching vibration at ~1730 cm^−1^ corresponding to O-acetyl esters, and C–O–C/C–O stretching between 1200 and 1000 cm^−1^ from mannose- and glucose-based polysaccharide backbones [33,37]. The band at 1730 cm^−1^ confirms the presence of acetylated mannan, a polysaccharide known to enhance bioactivity and wound healing [37]. The C=O and hydroxyl (–OH) vibrations reflect strong hydrogen-bonding capability, contributing to moisture retention and biological functionality [30,33,37]. SDS-PAGE of AV extract (Figure 1e) showed a distinct 35 kDa protein band, with additional bands in the 45–60 kDa range, indicating the presence of minor bioactive proteins and polysaccharide-protein complexes relevant for cell interaction and wound healing [37].

Our previous studies demonstrated that silk fibroin and *Aloe vera* gel extracts exhibit complementary interactions between the two biopolymers. Fibroin contributes a mechanically stable proteinaceous backbone and biological activities, such as the promotion of cell proliferation [24,30,37], while *Aloe vera* polysaccharides impart bioactive functionalities, including enhancement of cell proliferation and anti-inflammatory effects [33,37]. These synergistic interactions play a pivotal role in enhancing the wound-healing potential of the composite gel system.

### 2.2. Physicochemical Characteristics of Gel Formulations

The compositions of the base gels (B1–B5) and the extract-containing gel formulations (F1, F2, F5, and F6) are shown in Table 2. The ratios of AV, SFB, and CS in each sample are indicated, together with the ratios and concentrations (% *w*/*w*) of poloxamers used, including Poloxamer 188 (POL188) and Poloxamer 407 (POL407). All samples were formulated to maintain a total polymer content of 4% (*w*/*w*). Polyhexamethylene biguanide (PHMB) was incorporated at 0.1% (*w*/*w*) as an antimicrobial agent, and β-glycerophosphate (β-GP) was added at 2% (*w*/*w*), relative to the total polymer content, to aid gelation. Variations in the poloxamer ratios and concentrations were designed to investigate their effects on gelation properties and mechanical behavior.

#### 2.2.1. Rheology and Phase Transition Behavior of Thermoresponsive Gels Under Closed and Open Systems

**Figure 2:**
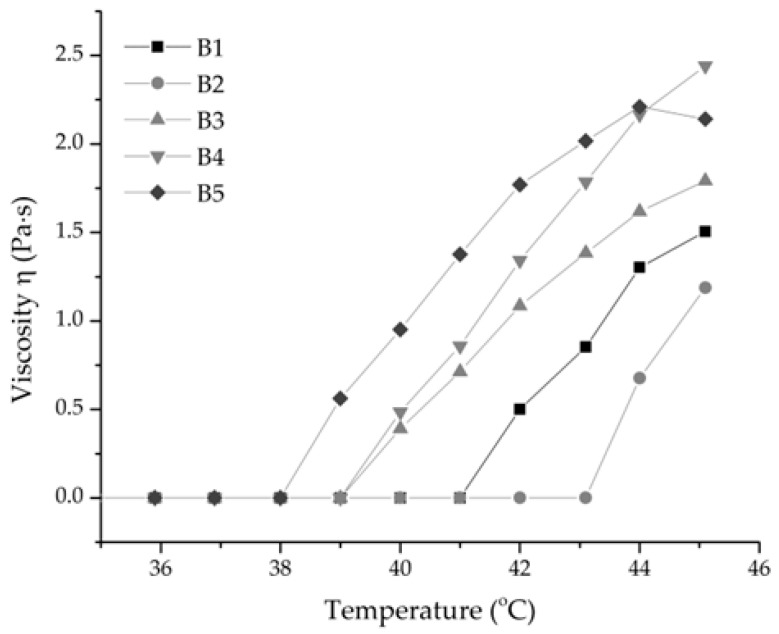
Temperature-dependent viscosity profiles of the gel formulations (B1–B5).

The temperature-dependent viscosity profiles of gel formulations B1 to B5 are presented in Figure 2. To select the optimal base gel formulation for further development containing silk fibroin and *Aloe vera* gel extract, the rheological behavior of each formulation was comparatively evaluated. The formulations demonstrated a distinct sol–gel transition, characterized by a sharp increase in viscosity in the range of 38–43 °C, corresponding to temperature-induced network formation typical of poloxamer-based systems [31,32]. At lower temperatures (36–39 °C), the viscosity remained close to zero, confirming that the samples were in a sol state.

Among the formulations, B5 displayed markedly higher viscosities with steeper transition profiles, reaching maximum values exceeding 2.0 Pa·s at approximately 45 °C. This behavior suggests that the increased proportion of POL407 promoted stronger gelation, whereas samples B2 and B3 showed intermediate rheological profiles, reflecting progressive improvements in gel strength with increasing POL407 content. This phenomenon can be explained by the unique properties of POL407, a triblock copolymer (PEO-PPO-PEO), which, together with the presence of CS and β-GP, leads to temperature-dependent micellization and gel formation [32]. Above the lower critical solution temperature (LCST), the hydrophobic PPO blocks aggregate to form micelles, which subsequently pack to create a three-dimensional gel network. [42,43,44]. The observed differences in viscosity behavior among B1 to B5 underscore the critical role of formulation composition, particularly poloxamer concentration, in modulating the rheological properties of the gels. B5 was selected as the most promising formulation for further development into bioactive formulations incorporating AV and SFB extracts (F): F5 (CS + POL188 + POL407, 10% *w*/*w* poloxamers) and F6 (CS + POL188 + POL407, 15% *w*/*w* poloxamers), with F1 (CS + POL188) and F2 (CS + POL407) serving as comparative controls, as shown in Table 1. The selection of B5 was based not only on its viscosity trend but also on its viscosity and viscoelastic properties, which allow easy spreading during application while providing sufficient structural resistance to prevent flow or loss from the wound surface. This links rheological thresholds to the practical requirements for topical gel application.

**Figure 3:**
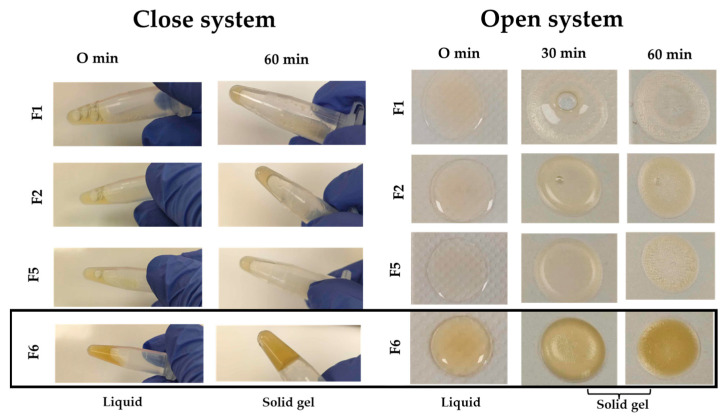
Visual observation of sol–gel phase transition behavior of the bioactive gel formulations (F1, F2, F5, and F6) at 0, 30, and 60 min under closed and open system conditions at 37 °C.

The thermoresponsive behavior (Figure 3) of the bioactive gel formulations was subsequently investigated at 37 °C under two experimental conditions: a closed system and an open system, in order to observe the phase transition from liquid to gel as a function of time. In the closed system, only F6 showed a clear sol–gel transition within 60 min, whereas F1, F2, and F5 remained mostly in the liquid state. In the open system, all formulations underwent gelation, with F6 transitioning the fastest. The observed differences between the two systems are attributed to moisture loss and heat transfer: open conditions promote water evaporation, increasing polymer concentration and accelerating micellization and network formation, whereas the closed system maintains high humidity, thereby limiting polymer aggregation and delaying gelation. These results indicate that the open system better represents practical wound application conditions.

**Figure 4:**
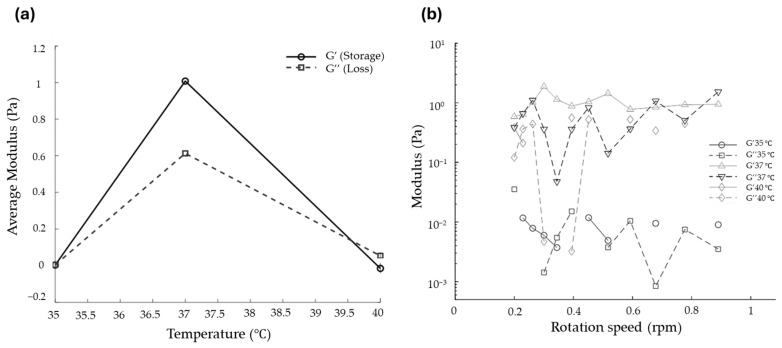
(**a**) G′–G″ crossover of F6 formulation, and (**b**) Relative modulus versus rotation speed for F6 formulations at 35 to 40 °C.

Further investigation of the thermoresponsive behavior of the F6 formulation was performed using dynamic oscillatory rheology in the low-shear range (0–1 rpm). As shown in Figure 4, the storage modulus (G′) and loss modulus (G″) exhibited clear temperature-dependent transitions. At 35 °C, both G′ and G″ remained near zero across the rotation range, indicating that the formulation was predominantly fluid-like, with minimal elastic network formation. At 37 °C, the gel demonstrated the highest viscoelastic strength, with G′ markedly increasing to approximately 1 Pa and surpassing G″ (~0.6 Pa), indicative of a dominant elastic network. The modulus values remained stable across the tested rotation speeds, consistent with the onset of a thermally driven sol–gel transition. At 40 °C, both moduli sharply decreased (G′ ≈ 0.05 Pa and G″ ≈ 0.1 Pa), indicating partial network destabilization at an elevated temperature. The reduction in G′ accompanied by an increase in G″ reflects a shift toward more viscous-dominant behavior. Averaged modulus data across the 0–1 rpm range further confirmed these observations: maximum G′ and G″ values occurred at 37 °C, with significantly lower values at 35 °C and 40 °C. The decrease in G′ observed at 40 °C is attributed to partial disruption and rearrangement of the physical micellar network at the elevated temperature. However, this temperature is higher than typical skin and wound surface temperatures (≈32–37 °C). Therefore, the observed drop in G′ is not expected to pose a practical limitation for topical wound application. The obtained thermoresponsive profile supports the suitability of the F6 gel formulation for physiological applications, where rapid in situ gelation and mechanical stability are critical for wound coverage and retention in diabetic wound microenvironments [31,39,40,41,42,43,44]. Therefore, the rheological data (G′, G″, temperature sweep) provide insight into gel stiffness, viscoelastic stability, and structural integrity under dynamic conditions. These parameters are directly relevant to clinical handling, including ease of spreading, retention on the wound site, and resistance to deformation, thereby justifying the use of rheology as a primary mechanical assessment in the absence of conventional tensile or compression testing.

#### 2.2.2. Crystal Structure and Surface Morphology of Base Gels and Gel Formulations

**Figure 5:**
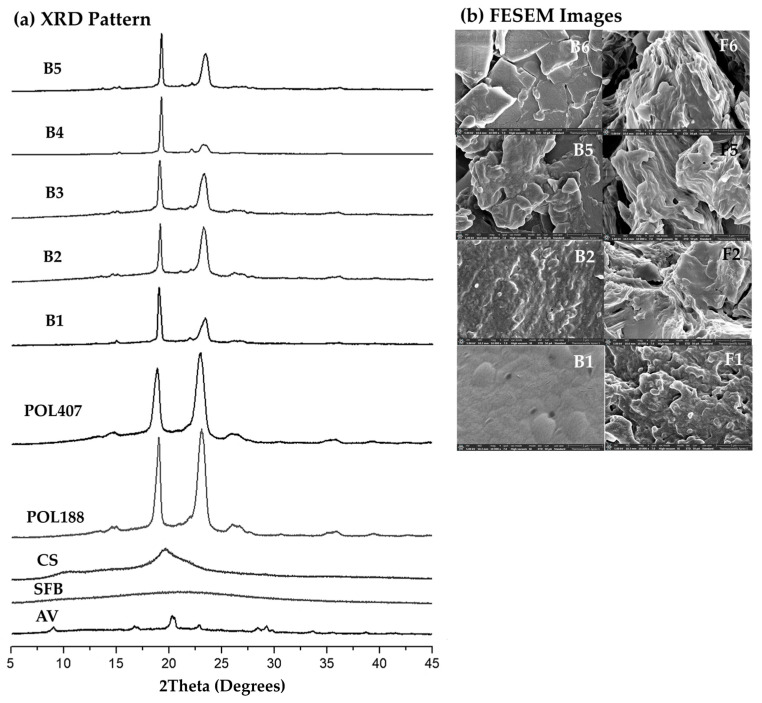
(**a**) X-ray diffraction (XRD) patterns of *Aloe vera* gel extract (AV), silk fibroin extract (SFB), chitosan (CS), Poloxamer 188 (PO-188), Poloxamer 407 (POL407), and gel base formulations (B1 to B5), and (**b**) FESEM images (10,000×) of gel base formulations (B1, B2, and B5), and bioactive formulations (F1, F2, F5, and F6) dried at 37 °C.

XRD patterns of the raw materials (Figure 5a) revealed distinct crystalline reflections for POL188 and POL407 at 2θ ≈ 19° and 23°, confirming their ordered molecular packing. Chitosan (CS) exhibited a broad reflection centered at 2θ ≈ 20°, consistent with a semi-crystalline structure arising from partial chain alignment and hydrogen bonding [37]. Silk fibroin (SFB) displayed a broad amorphous hump, whereas *Aloe vera* gel extracts (AV) presented weak diffuse signals with minor reflections between 2θ ≈ 15–30°, indicating a combination of semi-crystalline and amorphous components [37].

The XRD profiles of the base gel formulations (B1 to B5) displayed characteristic peaks at 2θ ≈ 20° and 23°. Notably, B1 showed low-intensity reflections, whereas B2 and B3 exhibited sharper peaks, suggesting increased crystallinity. B4 and B5 demonstrated the highest peak intensity with reduced full width at half maximum (FWHM), indicative of enhanced molecular ordering. These results confirm that crystallinity progressively increased with higher POL407 content. The variation in crystallinity among B1 to B5 is expected to directly influence their physicochemical performance, including mechanical strength and thermal stability [31,37]. The higher crystallinity observed in B5 suggests improved viscosity and structural stability, which are favorable characteristics for developing optimized bioactive gel formulations.

The surface morphology of the gel base formulations (B1, B2, and B5) that were further developed as bioactive gel formulations (F1, F2, F5, and F6) containing AV and SFB was examined using field emission scanning electron microscopy (FESEM), as shown in Figure 5b. A notable structural difference was observed between the base gels, which did not contain AV or SFB, and the F-series formulations, which incorporated equal amounts of CS, AV, SFB, PHMB, and β-GP, while varying only the ratio of POL188 and POL407. The base formulations displayed relatively smooth and compact surfaces with small, uniformly distributed pores, indicating a homogeneous polymeric structure. In contrast, the F-series formulations exhibited marked changes in internal microstructure following the incorporation of AV and SFB. These bioactive gels presented increased surface roughness with larger pore sizes, indicating altered polymer packing and cross-linking compared to the base gels.

Among the bioactive gel formulations, distinct morphological features were observed depending on the poloxamer composition. F1 (100% POL188) exhibited a uniformly porous structure with fine, well-distributed pores and a relatively smooth surface. F2 (100% POL407) displayed larger, sponge-like pores with a rougher surface texture. F5 and F6 (POL188: POL407, 20:80) presented a fiber-like arrangement with widely distributed pores, representing an intermediate morphology between F1 and F2. These morphological variations can be attributed to the distinct physicochemical properties of the poloxamers. Again, POL407, which possesses a higher molecular weight and longer hydrophobic polypropylene oxide (PPO) blocks, forms larger hydrophobic domains and enhances porosity through the development of more extensive micellar aggregates [32,43]. Conversely, POL188, with shorter PPO segments and a more balanced hydrophilic-hydrophobic character [42,43], promotes denser and smoother gel matrices. Therefore, formulations combining both poloxamers (F5 and F6) achieved a structural balance between compactness and porosity.

Moreover, CS, SFB, and AV contain reactive functional groups, including amino (–NH_2_), hydroxyl (–OH), and carboxyl (–COOH) groups, which are capable of forming physical or chemical crosslinks with β-GP [32,33,37]. These interactions generate polymeric networks with distinct densities and degrees of crosslinking, leading to alterations in gel microstructure, mechanical strength, rheological behavior, and gel contraction characteristics. The resulting more open, porous matrix may facilitate wound exudate management, enable controlled release of bioactive compounds at the wound site, and promote cellular responses, thereby enhancing the overall therapeutic efficacy of the formulations [37,39,40,41,42].

#### 2.2.3. Chemical Analysis of Base Gel and Gel Formulations by FTIR

**Figure 6:**
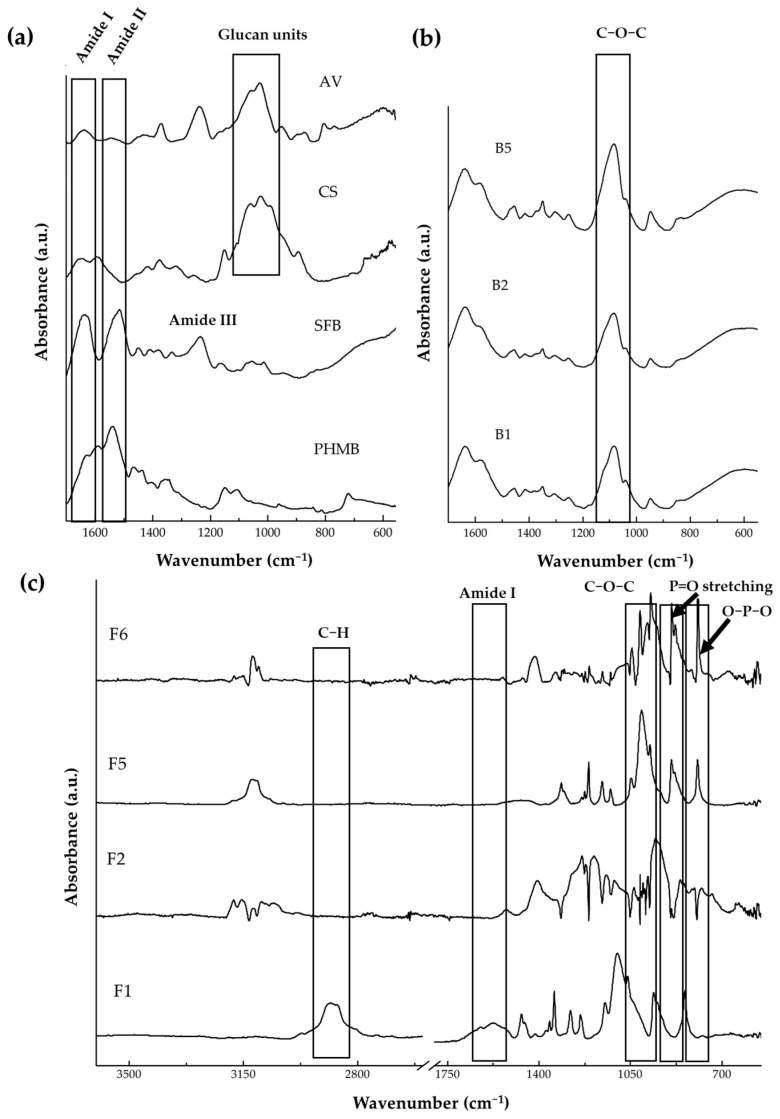
FTIR spectra of the gel formulations: (**a**) chitosan and PHMB, (**b**) base gels B1–B5 showing characteristic absorption peaks, and (**c**) bioactive gel formulations F1–F6.

As shown in Figure 6, all samples displayed characteristic absorption bands corresponding to polysaccharides and protein-based components, indicating the presence of key functional groups such as O–H, N–H, C–H, C=O, and C–O–C vibrations.

B1 (containing 100% POL188) and B2 (containing 100% POL407) exhibited characteristic absorption bands corresponding to the functional groups of each poloxamer. Specifically, both POL188 and POL407 showed distinct peaks in the region of ~1050–1150 cm^−1^ associated with C–O–C stretching vibrations of the ether linkages in the polyethylene oxide (PEO) and polypropylene oxide (PPO) blocks [42,43]. B5, prepared with a mixed POL188: POL407 ratio of 20:80, showed a more pronounced and broader C–O–C stretching peak in the ~1050–1150 cm^−1^ region. This enhancement may arise from the synergistic contribution of both poloxamers, where the higher proportion of POL407 (which has a higher molecular weight and longer PEO chains) increases the overall ether group density in the formulation. Additionally, the different chain lengths and hydrophilic-lipophilic balance (HLB) of the two poloxamers may lead to improved molecular packing and stronger intermolecular interactions, resulting in a more intense C–O–C absorption signal [43,44].

A key observation is the presence of the amide I band (~1640–1650 cm^−1^) in the bioactive gel formulations, particularly in F6. This peak is directly associated with β-sheet and random coil conformations of silk fibroin, as mentioned previously. Strong absorptions in the 1100–950 cm^−1^ region correspond to C–O–C and C–O stretching vibrations typical of AV and poloxamers, indicating hydrogen bonding interactions and successful blending of components [37,38,39]. Poloxamer incorporation further broadened the C–O–C region, reflecting molecular mixing and hydrogen bonding within the polysaccharide-polymer network.

Moreover, β-GP interactions were evidenced by the presence of bands at 900-800 cm^−1^ (P–O and O–P–O bending) and a decrease in intensity of the amide II (~1530 cm^−1^) and amide III (~1230 cm^−1^) bands, consistent with phosphate-mediated crosslinking between amino groups and β-GP. The most pronounced spectral changes were observed in F6, suggesting enhanced molecular ordering and a more stable hydrogel network, which contributes to improved thermoresponsive behavior, as mentioned earlier, and may also enhance cell adhesion.

### 2.3. Cytotoxicity Effects on Normal and Diabetic Fibroblasts

**Figure 7:**
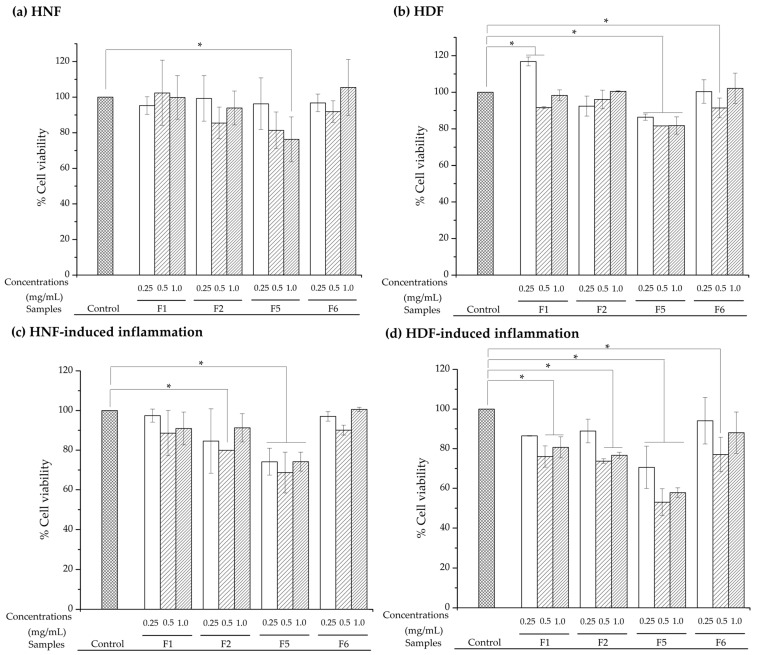
Percentage of cell viability of (**a**) normal skin fibroblast, (**b**) human diabetic fibroblast, (**c**) inflammation-induced fibroblasts, and (**d**) inflammation-induced human diabetic fibroblast treated with supernatants from formulations F1–F6 at concentrations of 0.25, 0.5, and 1 mg/mL, as determined by the MTT assay; Statistical significance was assessed by one-way ANOVA followed by Fisher LSD post hoc test. Asterisk indicate significant differences between groups (* *p* < 0.05).

The biocompatibility of bioactive gel formulations was evaluated using HNF and HDF under both normal and inflammatory conditions. In vitro, an inflammatory diabetic fibroblast model was established using high glucose, LPS, IL-6, and TNF-α to simulate the complex microenvironment of diabetic wounds [34,35]. While this combination represents severe inflammatory conditions, it provides a stringent test for evaluating the cytocompatibility and bioactivity of the gel formulations. Under normal culture conditions (Figure 7a,b), formulations F1, F2, and F6 demonstrated excellent biocompatibility, with cell viability maintained above 80% across all tested concentrations (0.25, 0.5, and 1.0 mg/mL) in both cell types. This meets the ISO 10993-5 standard [45] for non-cytotoxic biomaterials, confirming the safety of the tested formulations for wound-healing applications. However, F5 at a concentration of 1.0 mg/mL showed viability below 80%, indicating potential cytotoxicity even under normal conditions.

**Figure 8:**
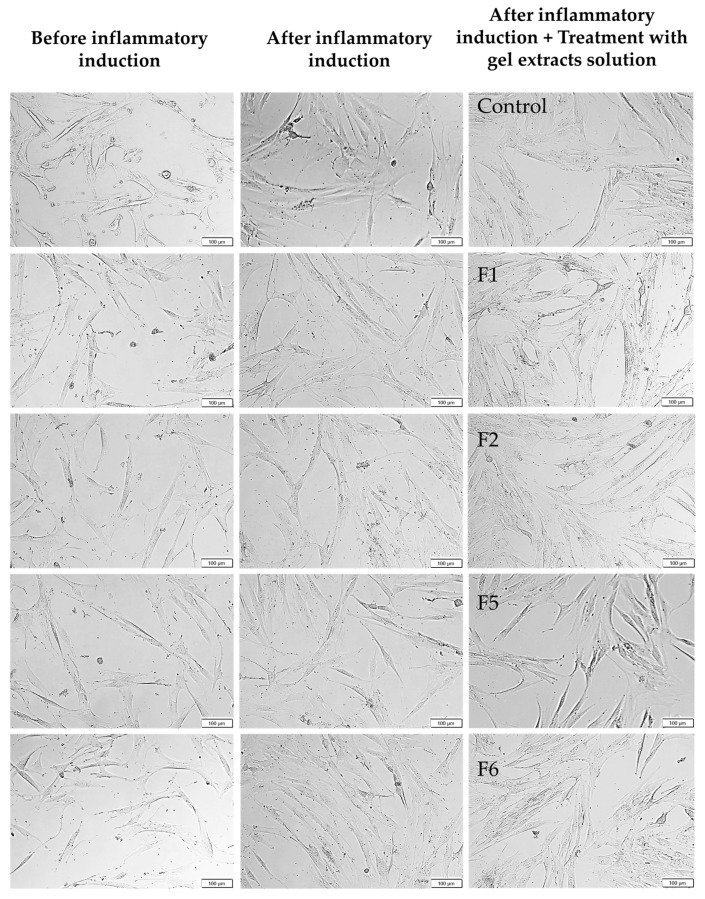
Representative cell morphology images of human normal fibroblasts (HNF) before and after inflammatory induction and after treatment with extract solutions of bioactive gel formulations F1, F2, F5, and F6 at a concentration of 1.0 mg/mL for 24 h, 10×.

**Figure 9:**
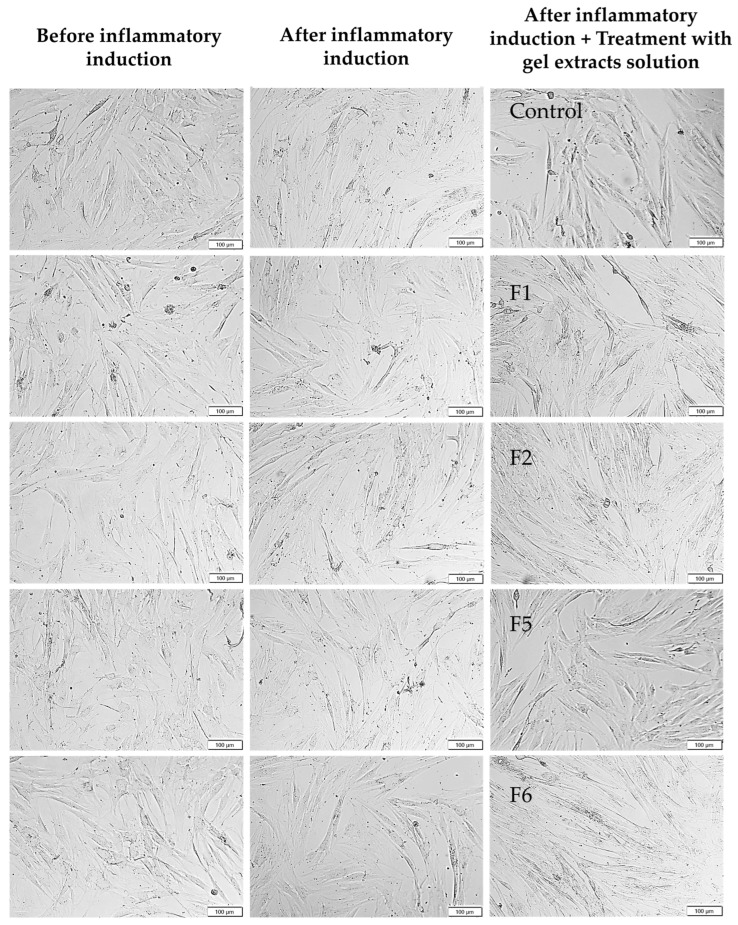
Representative cell morphology images of human diabetic fibroblasts (HDF) before and after inflammatory induction and after treatment with extract solutions of bioactive gel formulations F1, F2, F5, and F6 at a concentration of 1.0 mg/mL for 24 h. 10×.

Under inflammatory conditions mimicking the diabetic wound microenvironment, formulation-dependent differences in cellular responses became evident (Figure 7c,d). The inflammatory medium, containing high glucose (30 mM), LPS, IL-6, and TNF-α, induced cellular stress that typically impairs fibroblast viability and wound healing in diabetic conditions [34,35]. For HNF under inflammatory stress (Figure 7c), F5 (10% total poloxamer) at higher concentrations (0.5 and 1.0 mg/mL) showed reduced viability of 70-75%. Most notably, F6 (15% total poloxamer) maintained viability ≥ 80% at all tested concentrations, demonstrating non-cytotoxic properties. This phenomenon was also observed in HDF under inflammatory conditions (Figure 7d), where F6 again showed the highest cell viability (80–90%) across all concentrations, significantly outperforming F5, which exhibited pronounced cytotoxicity at higher concentrations (~55–70% viability). Examples of HNF and HDF morphology before and after inflammatory induction are shown in Figure 8 and Figure 9, respectively. The cellular morphology after treatment with gel extracts following inflammatory induction is also shown. HNF exhibited typical healthy fibroblast morphology with elongated, spindle-shaped structures and well-spread cytoplasm under normal conditions, while HDF displayed more variable morphology with less pronounced spindle-shaped characteristics. After inflammatory induction, both cell types showed signs of stress, including reduced cell spreading and rounded morphology. Treatment with gel extracts, particularly F6, maintained cell viability and adhesion to the culture surface, despite exposure to inflammatory stress. Under inflammatory conditions mimicking the diabetic wound microenvironment, formulation-dependent differences in cellular responses became evident (Figure 7c,d).

The exceptional performance of F6 can be attributed to the critical role of total poloxamer concentration in maintaining gel network integrity and protective capacity under inflammatory stress. Both F5 and F6 share identical bioactive compositions (AV:SFB:CS = 1:36:155) and the same poloxamer ratio (POL188:POL407, 20:80), with the key difference being total poloxamer content: 10% (*w*/*w*) in F5 versus 15% (*w*/*w*) in F6. As evidenced by previous rheological and structural analyses, the higher poloxamer content in F6 creates a more robust and stable hydrogel network than F5. FTIR spectra also revealed that F6 exhibited more pronounced spectral changes, suggesting stronger hydrogen bonding networks between poloxamers and bioactive components. The difference in network density between F5 and F6 may influence the diffusion and retention of leachable components during the extraction period. The looser network structure of F5 could allow a higher diffusion of soluble or weakly associated components into the extraction medium, whereas the denser network of F6 (15% poloxamer) is likely to retain formulation components more effectively within the gel matrix. Therefore, the different biological responses observed between F5 and F6 are more likely associated with differences in formulation composition and network structure. The higher poloxamer concentration also promotes more complete micellization and network formation, reducing the amount of free, unassociated poloxamer chains that could contribute to osmotic stress in the extract, thereby reducing cytotoxicity to cells.

These findings establish that F6, with its optimized 15% total poloxamer content, represents the most promising formulation for diabetic wound-healing applications. The increased poloxamer concentration provides not only superior structural integrity and thermoresponsive properties (as demonstrated previously) but also essential protective capacity that distinguishes it from F5. The ability of F6 to maintain ≥ 80% cell viability across all tested concentrations under these simulated diabetic conditions demonstrates its potential to support critical cellular processes, including migration, proliferation, and extracellular matrix synthesis, even in hostile microenvironments.

### 2.4. Fibroblast Migration in Response to Gel Formulations 

**Figure 10:**
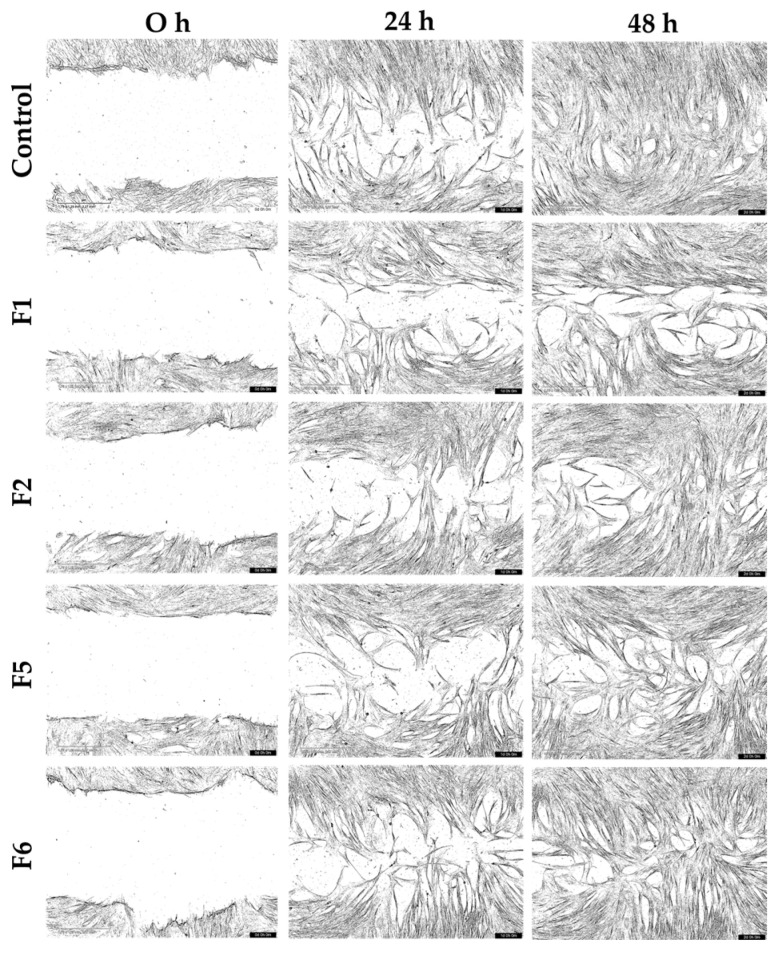
Representative scratch assay images showing migration of human normal fibroblasts (HNF) in normal medium treated with gel extract solutions from formulations F1, F2, F5, and F6 at a concentration of 1.0 mg/mL.

**Figure 11:**
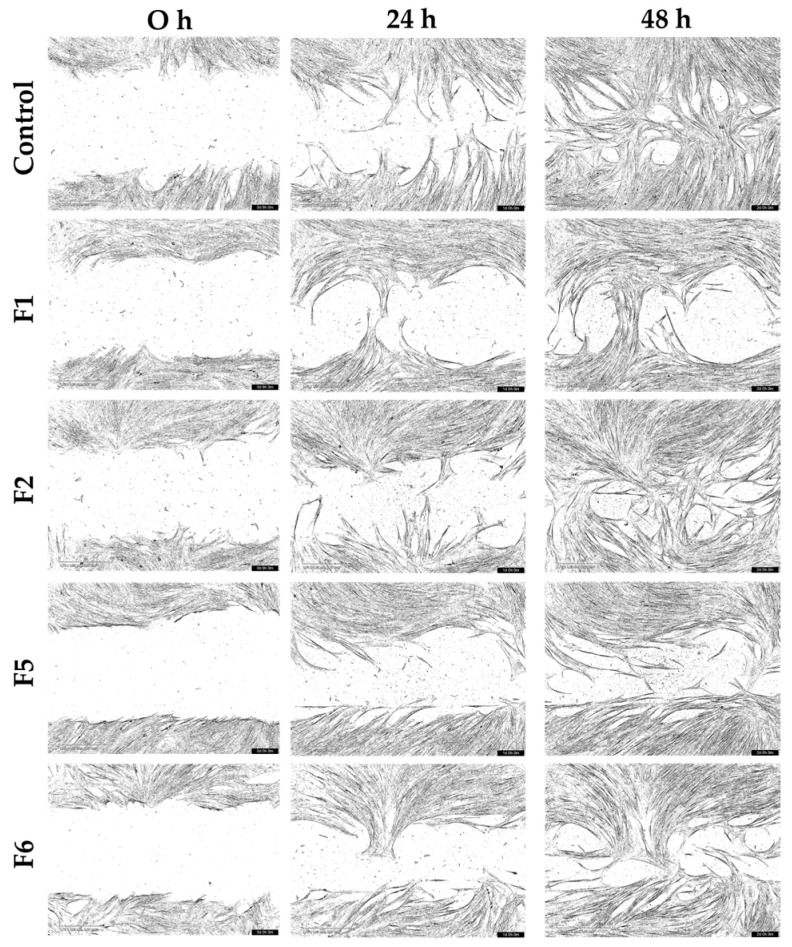
Representative scratch assay images showing migration of human normal fibroblasts (HNF) in an inflammatory condition treated with gel extract solutions from formulations F1, F2, F5, and F6 at a concentration of 1.0 mg/mL.

**Figure 12:**
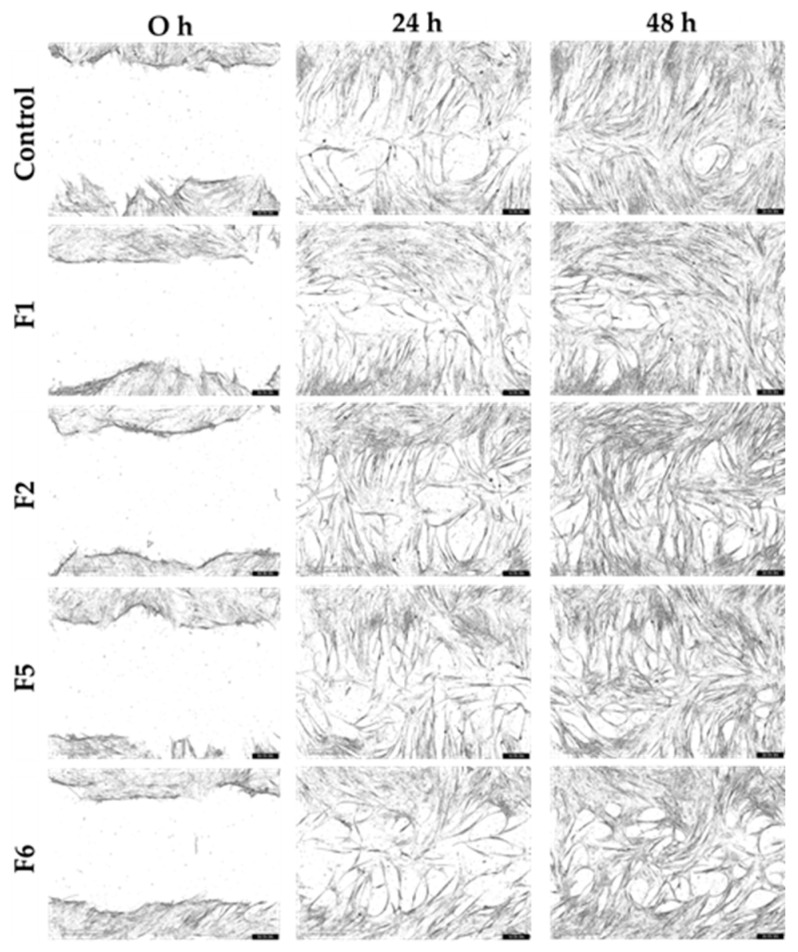
Representative scratch assay images showing migration of human diabetic fibroblasts (HDF) in normal medium treated with gel extract solutions from formulations F1, F2, F5, and F6 at a concentration of 1.0 mg/mL.

**Figure 13:**
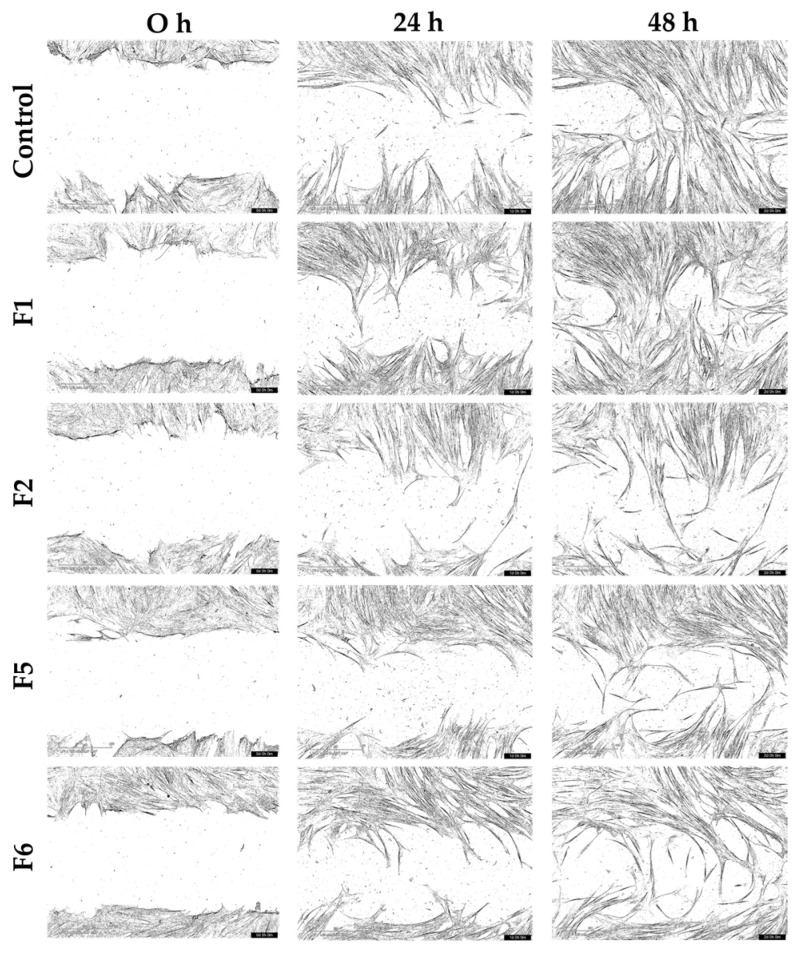
Representative scratch assay images showing migration of human diabetic fibroblasts (HDF) in an inflammatory condition treated with gel extract solutions from formulations F1, F2, F5, and F6 at a concentration of 1.0 mg/mL.

**Figure 14:**
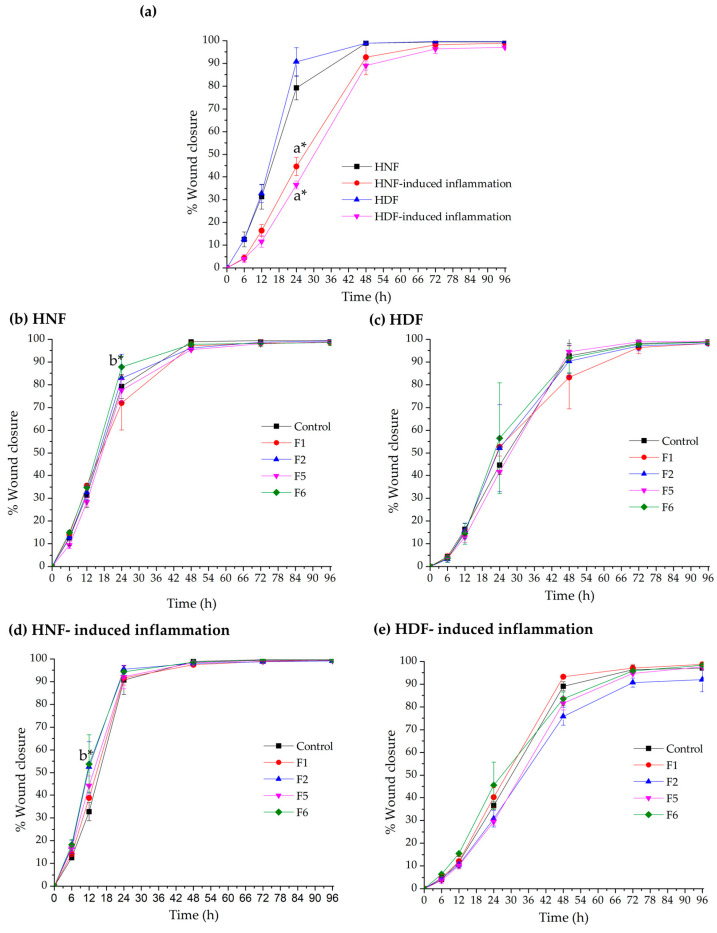
Wound closure percentage of (**a**) human normal fibroblasts (HNF) and human diabetic fibroblasts (HDF) under normal and inflammatory conditions, (**b**) HNF under normal condition, (**c**) HNF under inflammatory condition, (**d**) HDF under normal condition, and (**e**) HDF under inflammatory condition treated with extract solutions of bioactive gel formulations F1, F2, F5, and F6 at a concentration of 1.0 mg/mL. a* *p* < 0.05 comparing healing percentage between inflammatory and normal conditions; b* *p* < 0.05 compared to control for F6.

The wound closure capacity of the bioactive gel formulations (F1, F2, F5, and F6) was evaluated using scratch assays with HNF and HDF cultured under normal and inflammatory conditions. Representative migration images from the starting point (0 h) to 48 h are shown in Figure 10, Figure 11, Figure 12 and Figure 13, and the percentage of wound closure for HNF and HDF under normal and inflammatory conditions, with and without treatment with gel extract solutions, is presented in Figure 14.

Under inflammatory induction, the wound closure percentage, indicating the migration capacity of both HNF and HDF, was significantly reduced compared to normal culture conditions (*p* < 0.05, Figure 14a). This inhibitory effect was particularly pronounced in HDF under inflammatory conditions, demonstrating the synergistic impact of diabetes and inflammation on cellular migration. This combined pathological state is a major contributor to chronic non-healing wounds in diabetic patients. Therefore, gel formulations capable of enhancing cellular migration under these adverse conditions are expected to accelerate wound closure and reduce wound area effectively.

Compared to the control group (cells without gel extract treatment), F6 extract solution demonstrated statistically significant enhancement of wound closure in HNF. Specifically, significant differences were observed in HNF under normal conditions at 24 h (*p* < 0.05, Figure 14b) and in HNF under inflammatory conditions at 12 h (*p* < 0.05, Figure 14d). These findings indicate that bioactive components of F6 effectively promote early-stage migration in normal fibroblasts, regardless of the inflammatory environment. The significant effect at 12 h in inflamed HNF is particularly noteworthy, as this represents the critical early healing phase where rapid cellular response is essential for preventing wound chronicity. F6 treatment appeared to counteract the inflammation-mediated delay in migration, enabling cells to initiate wound closure more quickly even under inflammatory conditions.

While statistical significance was not achieved in HDF, F6 treatment showed a consistent trend toward improved wound closure capacity compared to the control in both normal and inflammatory conditions at 24 h (Figure 14c,e). This trend suggests potential therapeutic benefit, though the effect may be more subtle in diabetic cells due to their inherently compromised cellular machinery. The lack of statistical significance in HDF may result from the more severe cellular dysfunction in diabetic fibroblasts, which may require higher concentrations or prolonged exposure to bioactive compounds. Despite not reaching statistical significance, the consistent positive trend across both conditions suggests that F6 may still provide clinical benefit for diabetic wound healing, warranting further investigation with optimized treatment protocols. It should be noted that, although some treatments showed an apparent increase in HDF migration, several comparisons did not reach statistical significance and should therefore be interpreted as observed trends rather than definitive effects.

The differential response between HNF and HDF to F6 treatment provides important insights into wound healing dynamics. The significant early-stage enhancement in HNF, particularly under inflammatory conditions, suggests that F6 can effectively overcome inflammation-induced migration barriers in cells with intact baseline function. This finding is clinically relevant because even in diabetic wounds, the wound margin contains a mixture of normal and diabetic fibroblasts, and enhancing the migration of the normal cell population could still contribute to overall healing.

The observed trends in HDF, while not statistically significant, suggest partial effectiveness that could be further enhanced. through formulation optimization, such as adjusting bioactive concentrations or developing sustained-release systems to maintain therapeutic levels longer. Future studies should explore dose–response relationships specifically in HDF and investigate whether prolonged treatment durations could achieve statistically significant improvements in this challenging cell population.

## 3. Conclusions

In this study, a multifunctional thermoresponsive gel incorporating Silk fibroin (SFB) and *Aloe vera* gel extract (AV) was successfully developed and characterized. The F6 formulation, composed of POL188: POL407 at a 20:80 ratio with a total poloxamer content of 15% (*w*/*w*), exhibited favorable thermoresponsive behavior, a stable three-dimensional network, and enhanced pore structure. In vitro evaluations demonstrated that F6 promoted early-stage cell migration and was non-cytotoxic.

Based strictly on statistically supported findings, these results indicate that the developed gel has potential for application in diabetic wound treatment. The limitations of this study, including the use of an in vitro model and the absence of ex vivo human skin validation, should be considered when interpreting the findings.

## 4. Materials and Methods

### 4.1. Materials

Yellow silk cocoons (*Bombyx mori*) were received from a local sericulture unit, Phitsanulok, Thailand. Fresh *Aloe vera* (L.) Burm.f. leaves were collected from Phitsanulok, Thailand. Chitosan (CS, MW: 50–100 kDa; 80–95% degree of deacetylation) was purchased from Bio21 Co. Ltd., Samutsakhon, Thailand. β-glycerophosphate (β-GP), sodium carbonate, ammonium sulfate, lactic acid, and polyhexamethylene biguanide (PHMB) were purchased from Sigma-Aldrich, St. Louis, MO, USA. Poloxamer 188 (POL188) and Poloxamer 407 (Pluronic F-127, POL407) were obtained from Sigma-Aldrich, USA. All other reagents were of analytical grade. Human normal fibroblasts (HNF) and human diabetic fibroblasts (HDF) were purchased from PELOBiotech GmbH, Planegg-Martinsried, Germany. Dulbecco’s Modified Eagle’s Medium (DMEM), fetal bovine serum (FBS), penicillin-streptomycin (PS, 10,000 U/mL), 0.05% trypsin/0.02% EDTA, and Dulbecco’s phosphate-buffered saline (DPBS) were purchased from PANBiotech, Aidenbach, Germany. Thiazolyl blue tetrazolium bromide (MTT reagent) and dimethyl sulfoxide (DMSO) were purchased from Sigma-Aldrich, USA. Lipopolysaccharide (LPS), interleukin-6 (IL-6), and tumor necrosis factor-α (TNF-α) were purchased from Thermo Fisher, Illkirch-Cedex, France.

### 4.2. Silk Fibroin Extraction

Silk fibroin was extracted following a previous method [33]. Briefly, B. mori silk fibers were degummed in NaOH solution to remove sericin, rinsed thoroughly with deionized water, and air-dried. The treated fibers were dissolved in a 6 M CaCl_2_ solution. The resulting solution was dialyzed against deionized water using dialysis tubing to remove salt, then centrifuged to remove insoluble particles. The supernatant was collected and lyophilized for further experiments.

### 4.3. Aloe vera *(L.) Burm.f.* Extraction

Fresh *Aloe vera* leaves were manually peeled to isolate the inner gel, which was homogenized. Proteins and bioactive compounds were precipitated using ammonium sulfate ((NH_4_)_2_SO_4_). The precipitate was dissolved in deionized water with stirring, followed by dialysis against deionized water for desalting. The dialyzed solution was freeze-dried, and the lyophilized powder was kept for subsequent experiments [33].

### 4.4. Characterization of the Extracts

#### 4.4.1. Protein Content

Silk fibroin and *Aloe vera* gel extracts were quantified using the Detergent-Compatible (DC) protein assay kit (Lowry method), Millipore, Merck, Germany. Briefly, samples were diluted appropriately and mixed with DC reagents according to the manufacturer’s instructions. The absorbance was measured at 750 nm using a microplate reader, and protein concentrations were determined from a standard curve prepared with bovine serum albumin (BSA).

#### 4.4.2. Molecular Weight Distribution

The molecular weight distribution of the extracts was determined by Sodium Dodecyl Sulfate Polyacrylamide Gel Electrophoresis (SDS-PAGE). Samples were denatured and loaded onto polyacrylamide gels. After electrophoresis, gels were stained with Coomassie Brilliant Blue, and protein bands were visualized against a standard protein marker.

#### 4.4.3. Chemical Analysis

The chemical functional groups of both extracts were identified using an Attenuated Total Reflectance-Fourier Transform Infrared (ATR-FTIR) Spectroscopy (Frontier, PerkinElmer, Waltham, MA, USA). Spectra were collected at 2 cm^−1^ resolution with 64 scans over the wavenumber range of 4000–400 cm^−1^. The resulting spectra were analyzed to identify characteristic absorption bands of silk fibroin and *Aloe vera* extracts.

Moreover, curve fitting of the amide I region using a Gaussian function further confirmed the presence and proportion of these secondary structural components (Figure 1b).

(1) $$y=y_{0}+\frac{A}{w\times \sqrt{4In2}}\times exp[-4In(2)\times {\left(\frac{x-x_{c}}{w}\right)}^{2}]$$

whereas y_0_ = baseline offset (background intensity), A = area under the peak, w = peak width (related to full width at half maximum), and x_c_ = peak center position that provides the Curve-fitted amide I region (1700–1600 cm^−1^) after baseline correction and second-derivative guidance; Component peaks were modeled with Gaussian-Lorentzian functions.

### 4.5. Formulation of Thermoresponsive Gel Incorporating Silk Fibroin and Aloe vera Extracts

The thermoresponsive gel was prepared through a three-step process: (i) preparation of a polymeric base, (ii) incorporation of *Aloe vera* (AV) and silk fibroin (SFB) extracts, and (iii) addition of antimicrobial and gel-inducing agents. The combined concentration of AV, SFB, and chitosan (CS) ranged from 4% *w*/*w*, with the total poloxamer content from 10–15% *w*/*w*. The final pH of the gel was maintained within 5.5-7.0.

POL188 and POL407 were combined at weight ratios of 100:0, 0:100, 50:50, 30:70, and 20:80, and then mixed with a fixed amount of chitosan. Polymer mixtures were dissolved in 0.5 M lactic acid under continuous stirring until a homogeneous solution was obtained. Deionized water (DI) was subsequently added to adjust the solvent composition, followed by slow neutralization using 10% *w*/*v* sodium bicarbonate solution to partially raise the pH and stabilize the polymer matrix prior to bioactive incorporation.

For incorporating AV and SFB into the pre-neutralized poloxamer-CS solution, AV and SFB extracts were prepared separately at a fixed AV: SFB weight ratio of 1:36, as previously reported [33]. Each extract was dissolved separately in DI water and gently mixed until complete dissolution. The AV-SFB mixture was then slowly added to the poloxamer-CS solution under gentle stirring for homogeneous dispersion.

Polyhexamethylene biguanide (PHMB) and β-glycerophosphate (β-GP) were separately dissolved in DI water and subsequently added to the polymer-bioactive mixture and stirred for 0.5–2 h until fully homogeneous.

To eliminate entrapped air bubbles, the final formulation was subjected to ultrasonication and aseptically transferred into containers. Gels were stored at 4 °C for 24–48 h for stabilization and network formation. The resulting thermoresponsive bioactive gel was subsequently used for physicochemical characterization and biological evaluation. The prepared formulations are shown in Table 2.

### 4.6. Physicochemical Characterization of Gel Formulations

#### 4.6.1. Phase Transition and Gelation Time

In the closed system, 250 µL of gel solution was placed in vials and heated gradually in a water bath. The sol-to-gel transition temperature was identified by the vial inversion method. When the sample no longer flowed upon inversion. For gelation time measurement, samples were transferred directly to a 37 °C water bath, and the time to reach the non-flowing state was recorded.

In the open system, 75 µL of gel solution was dropped onto glass slides and placed in a hot air oven at 37 °C. Gelation time was determined when the droplet ceased to flow upon tilting the slide. All measurements were performed in triplicate [30].

#### 4.6.2. Rheological Analysis

Rheological properties were measured to assess in situ gelation of the gel. Tensile, compressive, and adhesion tests were not performed in this study. Gel formulations were analyzed using a rheometer (RST, Brookfield Ametek, Middleboro, MA, USA). Rheological measurements were performed using a rotational rheometer. Viscosity was measured in rotational mode over shear rates ranging from 1 to 100 s^−1^, while oscillatory tests were conducted in strain-controlled mode to determine the storage (G′) and loss (G″) moduli at 37 °C.

Temperature sweep tests were performed to determine the sol–gel transition behavior by heating samples from 25 to 45 °C at a rate of 0.5 °C/min. The tests were conducted under oscillatory mode at 0.1% strain and 1 Hz frequency. The storage modulus (G′) and loss modulus (G″) were recorded as functions of temperature. The sol–gel transition temperature (T_sol-gel) was defined as the crossover point where G′ = G″. Data analysis and determination of the crossover point were performed using MATLAB version R2025a (MathWorks, Natick, MA, USA).

#### 4.6.3. Morphological Analysis

The surface morphology of the gel samples was examined using Field Emission Scanning Electron Microscopy (FESEM, Apreo 5, Thermo Fisher Scientific, Waltham, MA, USA). Prior to analysis, gel samples were dried in a hot air oven at 37 °C until completely dehydrated. The dried samples were then mounted on aluminum stubs using double-sided carbon tape and sputter-coated with a thin layer of gold. FESEM images were captured to observe the surface structure.

#### 4.6.4. Crystalline Structure Analysis by X-Ray Diffraction (XRD)

The crystalline structure of the samples was characterized by X-ray diffraction (XRD) using a D2 PHASER diffractometer (Bruker, Billerica, MA, USA) equipped with Cu Kα radiation (λ = 1.5406 Å). The instrument was operated at 40 kV and 40 mA. Dried gel samples were finely ground into powder and uniformly loaded onto the sample holder to ensure a smooth and even surface. XRD patterns were recorded over a 2θ range of 5–50° with a step size of 0.02° and a counting time of 1 s per step at room temperature. The obtained diffraction patterns were analyzed to evaluate the crystallinity.

#### 4.6.5. Chemical Functional Groups

FTIR-ATR spectroscopy was performed to investigate the chemical interactions in the gel formulations using the same instrument and conditions described in the previous section. The spectra of gel formulations were compared with those of individual components to identify potential interactions, crosslinking, and structural changes during gel formation.

### 4.7. Evaluation of Gel Formulation Effects on Fibroblast Viability and Migration in Normal and Inflammatory Conditions

#### 4.7.1. Preparation of Test Samples

Gel samples at various amounts were extracted at 10 mg/mL in DMEM containing 5% FBS and 1% PS by incubation at 37 °C for 24 h. The extract was then filter-sterilized through a 0.2 µm membrane. The resulting extract was then serially diluted with culture medium (DMEM supplemented with 5% FBS and 1% PS) to achieve final gel concentrations of 0.25, 0.5, and 1.0 mg/mL for cell treatment.

#### 4.7.2. Culture Media Used

HNF and HDF were cultured under two conditions: (1) normal medium-DMEM with 5% FBS and 1% PS; and (2) inflammatory medium-normal medium supplemented with 30 mM glucose, 1 µg/mL LPS, 20 ng/mL IL-6, and 20 ng/mL TNF-α [34,35].

#### 4.7.3. Cytotoxicity

The cytotoxicity of the test samples was evaluated using the MTT assay [3-(4,5-dimethylthiazol-2-yl)-2,5-diphenyltetrazolium bromide] on HNF and HDF under both normal and inflammatory conditions. Cells were seeded at 1 × 10^4^ cells/well in 96-well plates and incubated for 24 h at 37 °C in 5% CO_2_ to allow attachment. After washing with PBS, cells were divided into two groups: (1) Normal conditions: Cells were incubated with normal medium (DMEM + 5% FBS + 1% PS) and (2) Inflammatory conditions: Cells were incubated with inflammatory medium (normal medium supplemented with 30 mM glucose, 1 µg/mL LPS, 20 ng/mL IL-6, and 20 ng/mL TNF-α). After 24 h of incubation, the medium was replaced with 100 µL of the test sample at various concentrations (equivalent amount of 0.25, 0.5, 1.0 mg gel/mL DMEM containing 5% FBS and 1% PS). Following incubation for 24 h, cells were rinsed with PBS and exposed to 0.5 mg/mL MTT solution (100 µL/well) for 4 h at 37 °C. The resulting formazan crystals were solubilized in 100 µL dimethyl sulfoxide (DMSO), and absorbance was recorded at 517 nm using a microplate reader. Cell viability was quantified as a percentage relative to the untreated control. Cell morphology was also observed by optical microscopy. All experiments were performed in triplicate.

#### 4.7.4. Cell Migration

Cell migration was evaluated using a scratch wound healing assay. HNF and HDF (2 × 10^5^ cells/well) were seeded in 96-well plates and incubated for 24 h at 37 °C in a humidified 5% CO_2_ atmosphere to allow cell adhesion and formation of a confluent monolayer. After washing with PBS, cells were assigned to 2 experimental groups. The inflammatory induction group was maintained with 100 µL culture medium. A linear scratch wound was then created on the cell monolayer using an IncuCyte^®^ Wound Maker tool (Sartorius, Heidelberg, Germany). Wells were washed twice with PBS to remove detached cells and debris. The test sample (100 µL of fresh gel extract solution at the designed concentration) was then added to each well. Cell migration was monitored from 0 to 96 h using an IncuCyte^®^ S3 live-cell imaging system (Sartorius, Germany) equipped with a 10× objective. Images were automatically captured every 3 h until wound closure.

Wound area was quantified using the Wound Healing Size Tool, an ImageJ (ImageJ 1.54-win, Java 8) [36]. The wound closure percentage was calculated according to the following equation:

(2) $$\mathrm{Wound}\;\mathrm{closure}\;(\%)=\frac{A_{1}-A_{2}}{A_{1}}\times 100\%$$

where A_1_ is the initial scratched area at 0 h, and A_2_ is the remaining wound area at each time point (3, 6, 12, 24, 48, 72, and 96 h). All experiments were performed in triplicate.

### 4.8. Statistical Analysis

Data are presented as mean ± standard deviation (SD) from three independent experiments. A limitation of this study is the small sample size (n = 3) used for the biological assays, which may reduce the statistical power of the results. Statistical significance was assessed using one-way analysis of variance (ANOVA) followed by Fisher’s LSD post hoc test at a 95% confidence level. Significance is indicated as *p* < 0.05, *p* < 0.01, and *p* < 0.001.

## 5. Patents

The experiment of gel formulations followed the petty patent number 2503002834 in the title of “The process of gel production for diabetic wound healing, incorporating Silk fibroin and *Aloe vera* gel extracts”, Department of Intellectual Property, Thailand (1 August 2025).

## Figures and Tables

**Table 1:** Protein content (%) of silk fibroin (SFB) and *Aloe vera* gel extracts (AV).

Extracts	Protein Contents (%)
SFB	93.00 ± 2.00
AV	7.00 ± 0.67

**Table 2:** Formulations of gel base (B) and thermoresponsive gel (F) incorporating *Aloe vera* gel (AV) and silk fibroin (SFB) extracts.

Samples	Ratios	Ratio of Poloxamers		Concentration of Poloxamers
	AV:SFB	POL188	POL407	%*w*/*w*
B1	0:0	100	-	10
F1	1:36	100	-	10
B2	0:0	-	100	10
F2	1:36	-	100	10
B3	0:0	50	50	10
B4	0:0	30	70	10
B5	0:0	20	80	10
F5	1:36	20	80	10
F6	1:36	20	80	15

Note: The concentrations of *Aloe vera* gel extracts (AV), silk fibroin (SFB), and chitosan (CS) were adjusted to 4% (*w*/*w*). Polyhexamethylene biguanide (PHMB) was added at 0.1% (*w*/*w*), while β-glycerophosphate (β-GP) was incorporated at 2% (*w*/*w*) relative to the total polymer content.

## Data Availability

Data is contained within the article.

## Abbreviations

The following abbreviations are used in this manuscript:

LPS	Lipopolysaccharide
TNF-α	Tumor Necrosis Factor-alpha
IL-6	Interleukin-6
*w*/*v*	weight/volume
*w*/*w*	weight/weight
PHMB	Polyhexamethylene Biguanide
β-GP	β-glycerophosphate
DMEM	Dulbecco’s Modified Eagle Medium
MTT	3-(4,5-dimethylthiazol-2-yl)-2,5-diphenyltetrazolium bromide
XRD	X-Ray Diffraction
FESEM	Field Emission Scanning Electron Microscopy
FTIR	Fourier Transform Infrared Spectroscopy
HNF	Normal Skin Fibroblast
HDF	Human Diabetic Fibroblast
